# Book Reviews

**Published:** 1898-02

**Authors:** 


### BOOK REVIEWS.

A System of Medicine. Vol. V. By many writers. Edited by Thomas Clifford Allbutt, M.D., LL.D., F.R.C.P., etc., etc. The McMillan Co., 66 Fifth Ave., N. Y. Pp. 880. Price, $5.

The editor, in his preface to this volume, states that he finds that the expected limits of this work must be extended by at least one volume. This cannot be looked upon as altogether sad news for the contributors when the excellence of the work is considered, and most of us prefer a book which is not too much condensed in style.

In this volume the anatomy, functions, and diseases of the liver are considered by Drs. William Hunter, Hale White, Andrew Davidson, Lafleur, Hawkins, John Thomson, and Mr. Mayo Robson. Dr. Fitz discusses diseases of the pancreas. In diseases of the kidneys, Dr. Rose Bradford deals with the general pathology of the renal functions, Professor MacAlister with nephroptosis, and Dr. Dickinson with diseases of the kidney characterized by albuminuria. To Dr. Henry Morris has been allotted all the other diseases of the kidneys. Drs. W. M. Ord, Hector Mackenzie and W. W. Ord write upon diseases of the thyroid gland ; Dr. H. D. Rolleston on diseases of the spleen, and of the suprarenal bodies; Dr. George R. Murray on Hodgkin's disease ; and Professors Allbutt and W. Pridgen Teale on scrofula. Obesity is discussed by Sir Dyce Duckworth. Only a portion of the diseases of the respiratory system are considered in this volume, and of these Dr. Ransom has the general pathology and the treatment of asphyxia, and Dr. Hector Mackenzie the physical signs of diseases of the lungs and heart. Diseases of the nose, pharynx and larynx are divided among Drs. de Havilland Hall, Greville MacDonald and Watson Williams and Sir Felix Semon, the two last writing in conjunction the greater part of this section.

This volume carries on the very high degree of excellence of its predecessors. Each subject is considered fully but succinctly by a physician of great reputation in his own field, and all have been gone over carefully by the learned editor of the System.

We again commend the book, with the utmost confidence, to all who are desirous of keeping themselves well versed in up-to-date medicine.

The outward form of this volume is, of course, similar to those preceding it, for which we have had only words of praise.

A. W. S.

---

TRAUMATIC INJURIES OF THE BRAIN AND ITS MEMBRANES. By Charles Phelps, M.D., Surgeon to Bellevue and St. Vincent's Hospitals, New York. With Forty-nine Illustrations. D. Appleton & Co., 72 Fifth Ave., New York. Price, cloth, $5.00.

The author says that this work is designed to be a concise and systematic exposition of the injuries which the brain suffers from external violence, a division of brain surgery which has the greatest practical importance and has received the least careful attention. It has been based almost exclusively upon a series of five hundred cases of recent occurrence.

Part I. discusses general traumatic lesions, beginning with a consideration of cranial fracture. Chapters in this section are devoted to the pathology, symptomatology, diagnosis, prognosis and treatment of cranial injuries. Part II. contains a special study of pistol-shot wounds of the head, in their medico-legal and surgical relations, with an abstract of the results of a series of experiments on the cadaver. This section contains 41 half-tone illustrations of pistol-shot wounds, inflicted at both short and long range. This portion of the book is exceedingly instructive from a medico-legal point of view. The work closes with the "Condensed Histories of 300 Intracranial Traumatisms Selected from a Series of 500 Original Cases," 225 of which were verified by necropsy.

While of course this work will be of special interest to surgeons, it will also be found of exceeding value to general practitioners, who will occasionally feel the urgent need of the aid to be derived from a wider clinical observation than their own opportunities have afforded. The author's clinical opportunities have certainly been ample, and he has admirably succeeded in analyzing them for the reader's use. We know of no better book of its kind.

PATHOLOGICAL TECHNIQUE. A Practical Manual for the Pathological Laboratory. By Frank Burr Mallory, A.M., M.D., and James Homer Wright, A.M., M.D. With one hundred and five illustrations. W. B. Saunders, 925 Walnut Street, Philadelphia.

The need for a single volume of convenient size, containing the methods of pathological, histological, and bacteriological work, has been recognized for some time. This work, therefore, will undoubtedly meet with the hearty approval of workers in these lines.

The authors have given us all the important methods in a volume of 383 pages.

Part I. treats of the methods of Post Mortem Examinations, and is sufficiently complete for thorough work.

Part II. treats of Bacteriological Examinations. It includes chapters on apparatus, culture media, examinations at autopsies, study of cultures, diagnosis, and clinical bacteriology.

The increasing importance of bacteriological work in medical work is everywhere recognized. Part II. will, therefore, be appreciated by the general practitioners, as it contains all essential methods, conveniently arranged for clinical work.

Part III. treats of Histological Methods, and will be of particular value to laboratory workers and advanced students. The sections on blood, parasites, and other clinical examinations will, however, prove of equal interest to the general practitioner.

The book deserves a place in the working library of every practitioner who desires to do work in any or all of these lines.

F. S. B.

---

A CLINICAL TEXT-BOOK OF SURGICAL DIAGNOSIS AND TREATMENT. For Practitioners and Students of Surgery and Medicine. By J. W. McDonald, M.D., of Minneapolis. Published by W. B. Saunders, Philadelphia. Price $5.00.

We do not know when we have seen a work more practical or more valuable to both the general practitioner and surgeon. It is a work of 781 pages, entertainingly written and beautifully gotten out by the publishers. It is thoroughly up to date. It treats of the diagnosis and treatment of every portion of the human anatomy and deals with each subject clearly and concisely, the whole work being beautifully illustrated in a large number of cases by photographs of original cases. There are several colored plates which add particular value to the work. The operative technique in most of the major operations is clearly given both in word explanation and illustrative cuts. Particular attention must be called to the descriptions given and illustrated of the various operations on the alimentary tract, hernia, the kidneys and bladder. In fact the work is splendid throughout and should certainly find a home in the libraries of every physician. The author has taken great pains to illustrate every appliance used in surgical treatment, and for a ready reference book we know no superior.

---

OUTLINES OF RURAL HYGIENE. For Physicians, Students, and Sanitarians. By Harvey B. Bashore, M.D., Inspector for the State Board of Health of Pennsylvania. Illustrated with twenty engravings. Pages vi–84. Extra cloth, 75 cents net. The F. A. Davis Co., Publishers, 1914–16 Cherry Street, Philadelphia.

The author believes that a work of this character is called for on account of the "almost absolute neglect of sanitary rules in districts outside of the great cities, and the absence of special attention to this branch of sanitation in larger and more elaborate treatises." It is the author's intention that this book shall aid in the diffusal of sanitary knowledge in rural districts. Chapters are devoted to water-supply, waste disposal, soil, habitations, disposal of the dead; with an appendix by Professor Smith, of Yale University, on the "Normal Distribution of Chlorine." The most important principles of normal sanitation appear to be well presented.

---

CLINICAL METHODS. A Guide to the Practical Study of Medicine. By Robert Hutchison, M.D., M.R.C.P., Demonstrator in Pathology, London, and Harry Rainy, M.A., F.R.C.P., Edinburgh. Lea Bros. & Co., 706 Sansom St., Philadelphia. Price, $3.

This book aims at "describing those methods of clinical investigation by the proper application of which a correct diagnosis can be arrived at." It emphasizes the importance of method in the examination and study of cases. The first chapter describes the methods of case-taking in general, and includes a general scheme for the investigation of medical cases. Separate chapters are devoted to the clinical examination of children, the examination of pathological fluids, and clinical bacteriology. There are 137 illustrations and eight colored plates.

The book will be very helpful to the busy doctor. It comprises in compact shape all the modern scientific methods of the clinical examination of patients, with full directions upon all procedures which may contribute to a proper diagnosis. The authors have had ample experience in both clinical work and teaching, the results of which they have made available to the active practitioner in this volume.

---

Elements of Latin. For the Use of Students of Medicine and Pharmacy. By Crothers and Bice. Published by F. A. Davis Company, 1916 Cherry St., Philadelphia. Price $1.25.

As a teacher of medicine for seventeen years I have found that one of the greatest needs of the student was just such a book. Since the war, in the South, many of the students with a fair English education, have not had the time to study the languages, hence are deprived of the great assistance supplied by Latin and Greek in the etymology of medical terms. This deficieny is especially noticeable in prescription writing.

A study of the little volume under review, in an easy and plain way, enables the average student to learn enough of Latin and Greek to supply the deficiency and make himself master of the proper use of medical terms derived from these languages. We recommend the book to all medical students. G. G. R.

---

Illustrated Skin Diseases, Atlas and Text-book. By Wm. S. Gottheil, M.D., Professor of Skin and Venereal Diseases, New York School of Clinical Medicine, etc., etc. New York, E. B. Treat & Co. Parts 10–13, inc.

The last parts of this work follow the style of those which preceded. The text is clear of excessive verbiage, and the illustrations are, for the most part, more lifelike than we have seen in any other work. The index is complete and the formulary full. A chapter is devoted to cosmetics—a new departure. The completed work makes a very desirable book.

---

Boericke and Tafel, of Philadelphia, have published a small book (pp. 96) upon *Saw Palmetto* (Sabal Serrulata), by Edwin M. Hale, M.D., giving the history, botany, chemistry, pharmacology, provings (homeopathic), clinical experience and therapeutic applications of this shrub. Its introduction into medicine is rightly attributed to Dr. J. B. Read, of Savannah. The booklet may be had from the publishers for fifty cents.

LIPPINCOTT'S MAGAZINE FOR JANUARY, 1898.

The complete novel in January issue of LIPPINCOTT's is "John Olmstead's Nephew," by Henry Willard French. A hurried marriage is necessary to secure some solid interests and defeat an enemy, and thereupon hangs a romance.

"Christmas Gold," a tale of Australia, by Owen Hall, and "Christmas Eve at Bilger's," which was in a Western mining camp, by Frank H. Sweet, celebrate the season. Why should Christmas stories appear a month ahead of Christmas? These are just in time.

Philip G. Hubert, Jr., tells at some length, but in a lively way, of "A Detective who Detected." He was a newspaper man; it was a literary crime he was set to detect, and while on the trail of it he found something else.

"Canuck and Raoul" were a Canadian boy and a horse, between whom a strong affection existed. Elizabeth Knowlton Carter is the narrator.

Calvin Dill Wilson writes pleasantly of a subject which he knows by heart, "The Eastern Shore of Maryland." "Irrigation from Under Ground," by John E. Bennett, takes the reader to the other end of the United States and gives much unfamiliar information about the arid regions, and how they may be made habitable and fertile.

Among "Some Botanic Gardens," George Ethelbert Walsh finds those of London and Paris most important, but gives brief mention to others in various parts of the world.

George Archie Stockwell seems to prove that the belief in "Wolf-Children," from Romulus and Remus on, is something more than a superstition. Oscar Herzburg finds a good deal to say about "Druggists, Ancient and Modern."

Under the heading, "To-day in the Bible," William Cecil Elam collects many familiar expressions, strokes of humor, observations and reflections on life and human nature, sharply pointed sayings, and what-not, which people often use without knowing their origin.

The old chronicler, "Froissart," is affectionately handled by Emily Stone Whiteley.

